# P04.10. Bipolar disorder and complementary medicine: current evidence, safety issues, and clinical considerations

**DOI:** 10.1186/1472-6882-12-S1-P280

**Published:** 2012-06-12

**Authors:** R Hoenders, J Sarris, J Lake

**Affiliations:** 1 Center for Integrative Psychiatry, Lentis, Groningen, Netherlands; 2 Department of Psychiatry, Faculty of Medicine, University of Melbourne, Melbourne, Australia; 3 Arizona Center for Integrative Medicine, Tucson, USA

## Purpose

Bipolar Disorder (BD) is a debilitating syndrome that is often undiagnosed and under-treated. Population surveys show that persons with BD often self-medicate with complementary, alternative medicine (CAM) or integrative therapies in spite of limited research evidence supporting their use. To date no review has focused specifically on non-conventional treatments for BD. Our objective was to present a comprehensive review of non-conventional (complementary and integrative) interventions examined in clinical trials on BD, and to offer provisional guidelines for the judicious integrative use of CAM in the management of BD.

## Methods

PubMed, CINAHL, Web of Science and Cochrane Library databases were searched for human clinical trials in English during mid 2010 using Bipolar Disorder and CAM therapy and CAM medicine search terms. Effect sizes (Cohen’s d) were also calculated where data were available.

## Results

Several positive high-quality studies on nutrients in combination with conventional mood stabilizers and antipsychotic medications in BD depression were identified, while branched-chain amino acids and magnesium were effective (small studies) in attenuating mania in BD. In the treatment of bipolar depression evidence was mixed regarding omega-3 fatty acids, while isolated studies provide provisional support for a multi-nutrient formula, n-acetyl cysteine, and L-tryptophan. In one study acupuncture was found to have favorable, but non-significant effects on mania and depression outcomes.

## Conclusion

Current evidence supports the integrative treatment of BD using combinations of mood stabilizers and select nutrients. Other CAM or integrative modalities used to treat BD have not been adequately explored to date, however some early findings are promising. Select CAM and integrative interventions add to established conventional treatment of BD and may be considered when formulating a treatment plan for patients diagnosed with BD. It is hoped that the safety issues and clinical considerations addressed in this paper may encourage the practice of safety-conscious and evidence-based integrative treatment of BD.

